# Supplementary material for: Controlling bimerons as skyrmion analogues by ferroelectric polarization in 2D van der Waals multiferroic heterostructures
Source: Nat Commun. 2020 Nov 23;11:5930. doi: 10.1038/s41467-020-19779-6 (PMC7683542; doi:10.1038/s41467-020-19779-6)
Supplement: Supplementary file 1 — Supplementary Information [file 41467_2020_19779_MOESM1_ESM.pdf]

Supplementary Information for:

“Controlling bimerons as skyrmion analogues by ferroelectric polarization in 2D van der Waals multiferroic heterostructures”

Part 1. Calculation for exchange coupling parameters and perpendicular magnetic anisotropy

Based on the calculated total energies of different magnetic configurations (Figure S1), the exchange coupling parameters can be derived by following equations:

$$E_{FM} = E_{other} - (6J_1 + 6J_2 + 6J_3)M^2 - KM^2 \quad (1)$$

$$E_{AFM} = E_{other} - (-6J_1 + 6J_2 + 6J_3)M^2 - KM^2 \quad (2)$$

$$E_{FIM1} = E_{other} - (-2J_2 + 6J_3)M^2 - KM^2 \quad (3)$$

$$E_{FIM2} = E_{other} - (6J_2 - 2J_3)M^2 - KM^2 \quad (4)$$

$$\Delta E_1 = E_{AFM} - E_{FM} = 12J_1M^2 \quad (5)$$

$$\Delta E_2 = E_{FIM1} - E_{FM} = (6J_1+8J_2)M^2 \quad (6)$$

$$\Delta E_3 = E_{FIM2} - E_{FM} = (6J_1+8J_3)M^2 \quad (7)$$

$$J_1 = \frac{\Delta E_1}{12M^2} \quad (8)$$

$$J_2 = \frac{2\Delta E_2 - \Delta E_1}{16M^2} \quad (9)$$

$$J_3 = \frac{2\Delta E_3 - \Delta E_1}{16M^2} \quad (10)$$

The perpendicular magnetic anisotropy is calculated by

$$K = \frac{E(100) - E(001)}{M^2} \tag{11}$$

where $E(100)$ and $E(001)$ are the total energies with magnetization directions parallel and perpendicular to the plane of LaCl film, respectively.

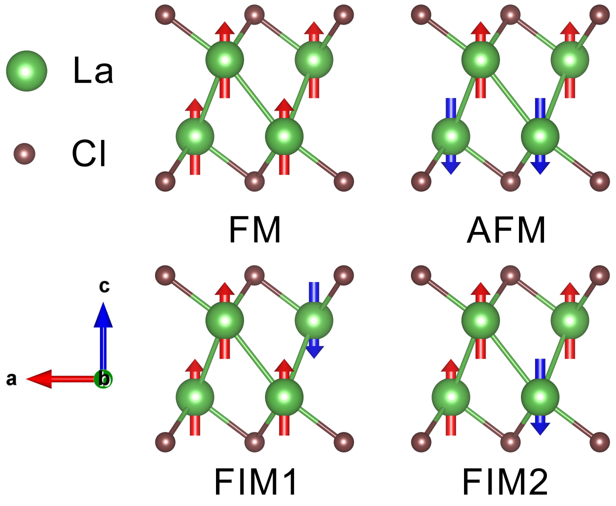

Fig. S1. **Four magnetic configurations for LaCl monolayer.** red and blue arrows are used to distinguish different magnetic directions.

Part 2. Calculation for DMI Vector

The DMI vector (**D**) is determined by mapping the total energies of artificially imposed spin configurations on the Hamiltonian: [1,2]

$$H = \sum_{i,j} \mathbf{D}_{i,j} \cdot (\mathbf{M}_i \times \mathbf{M}_j) \tag{12}$$

For the in-plane component D_x between the spin site 1 and spin site 2 ($\mathbf{M}_1$ and $\mathbf{M}_2$), calculated by setting the following four spin configurations: (i) $\mathbf{M}_1 = (0, \mathbf{M}, 0)$, $\mathbf{M}_2 = (0, 0, \mathbf{M})$, (ii) $\mathbf{M}_1 = (0, \mathbf{M}, 0)$, $\mathbf{M}_2 = (0, 0, -\mathbf{M})$, (iii) $\mathbf{M}_1 = (0, -\mathbf{M}, 0)$, $\mathbf{M}_2 = (0, 0, \mathbf{M})$, (iv) $\mathbf{M}_1 = (0, -\mathbf{M}, 0)$, $\mathbf{M}_2 = (0, 0, -\mathbf{M})$. In these four spin configurations, the spins of all the other spin sites are the same and are along the

z direction. The spin interaction energies of the four spin configurations were denoted as E_1, E_2, E_3, and E_4. The D_x can be calculated by:

$$D_x = \frac{E_1 + E_4 - E_2 - E_3}{4M^2} \tag{15}$$

Part 3. Calculation for Curie temperatures (T_c)

We performed Monte Carlo simulations with a 50 × 50 × 1 supercell based on the Heisenberg model to find the FM Curie temperature (T_c) of the LaCl monolayer and LaCl/In_2Se_3. For each temperature, 10^5 Monte Carlo updates were employed. The FM T_c is estimated by locating the peak position of the magnetic susceptibility, as shown in Fig. S2. Since we only calculated the DMI of LaCl/$P\pm$, the effects of the perpendicular magnetic anisotropy and exchange interaction terms on T_c was concerned in the Fig. S2a, and the effect of DMI on T_c is also concerned in LaCl/$P\pm$ in the Fig. S2b.

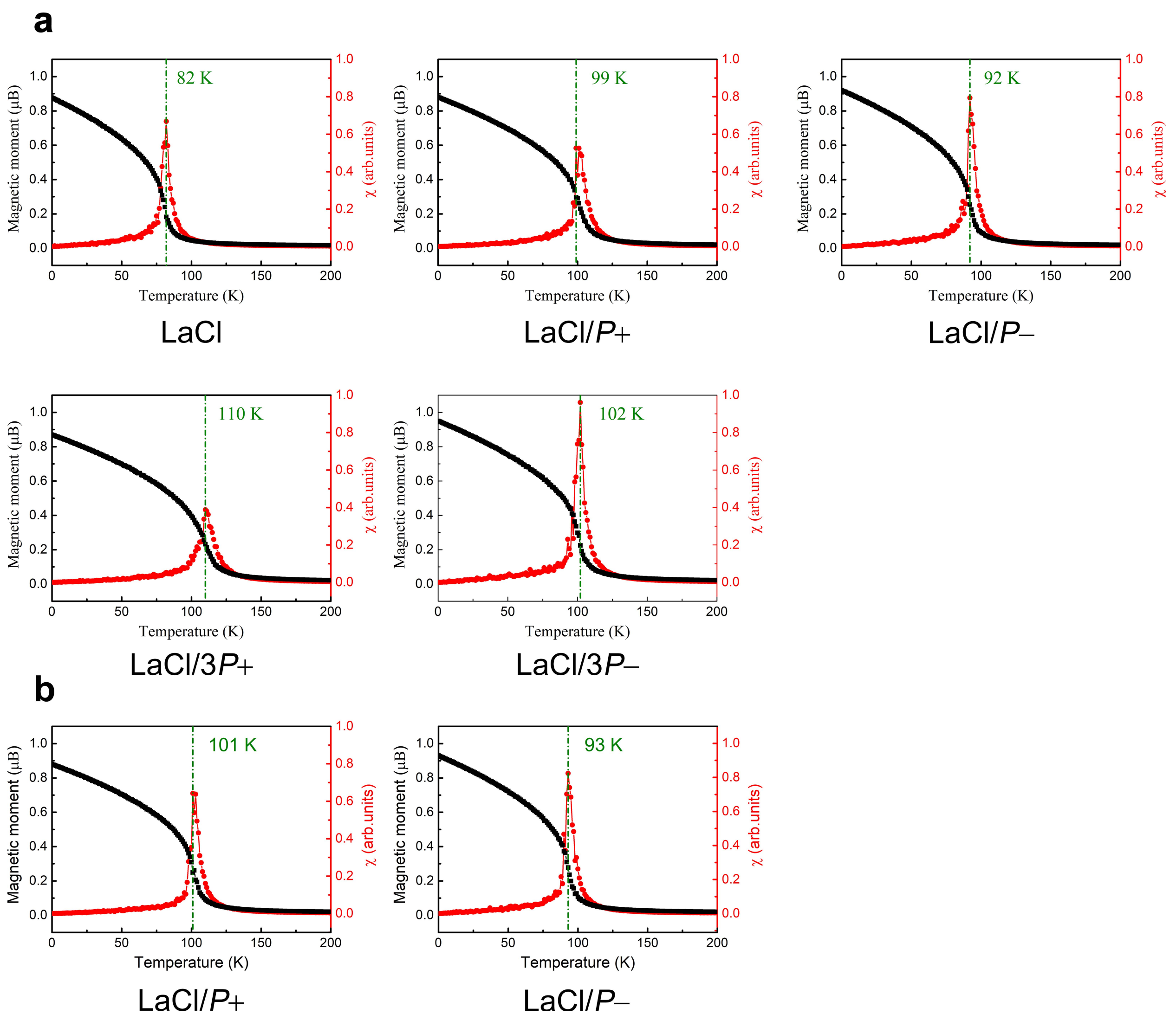

Fig. S2. The magnetic moment (black data) and magnetic susceptibility χ (red data) as functions of temperature for LaCl monolayer and LaCl/In_2Se_3 HSs. (a) The effect of DMI on temperature is not considered. (b) The effect of DMI on temperature is considered.

In our HSs, the T_c in (3)*P*+ state is higher than (3)*P*– state. Compared to the free-standing LaCl monolayer, the increase in the T_c mainly originate from the distinct increase of J_2 and J_3 exchange coupling under the (3)*P*+ state, due to the *K*, J_1, and magnetic moment are relatively insensitive to the polarization. In the (3)*P*– state, although J_1 is also enhanced, the values of J_2 and J_3, which are larger than J_1, are reduced. Therefore, the scenario of (3)*P*+ state is more conducive to the increase of the T_c. In addition, coordination number is also an important factor that needs to be considered, here, the larger *J* value with large coordination number leads to higher T_c. For any

one La ion, it will interact with three out-of-plane La ion and six in-plane La ion, as shown in Fig. S3. Combined with Fig.4a, the in-plane exchange coupling effect contributes more to T_c. In other words, the T_c in P+ state is higher than P– state.

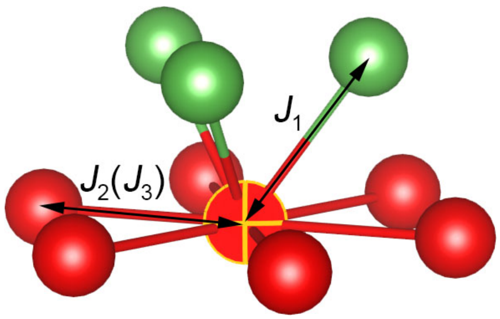

Fig. S3. The coordination number of La ion. same plane La ions are represented by the same color.

Part 4. Two bimerons with opposite topological numbers can coexist in nanodisk

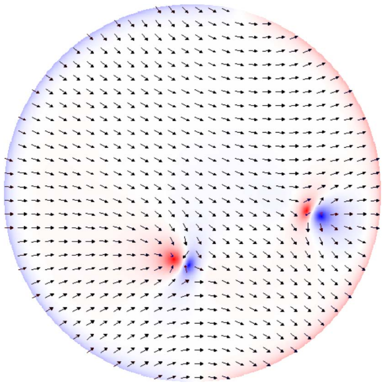

Fig. S4. The top views of the micromagnetic simulation for LaCl/*P*+ HS.

Part 5. The Relationship between bimeron size and **D**

In the nanodisk, the size of Néel-type skyrmions will increase with the increase of the **D** value [3]. However, whether this conclusion applies to bimeron is unknown. Here, we plot the bimeron size

as a function of **D** value, and other parameters are setting the same as LaCl/*P*+ scenario. It can be clearly seen from Fig. S5 that the size of bimeron indeed increases with the increase of **D** value.”

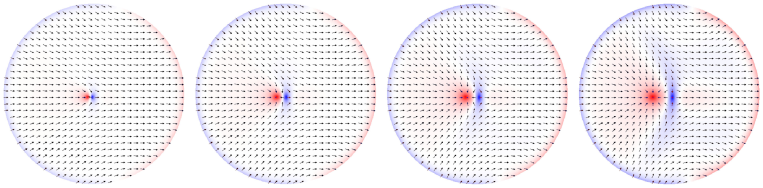

Fig. S5. The bimeron size in relation to D in the relaxed sample.

References

1. Xiang, H. J., Kan, E. J., Wei, S. H., Whangbo, M. H. & Gong, X. G. Predicting the spin-lattice order of frustrated systems from first principles. *Phys. Rev.* **84**, 224429 (2011).

2. Yang, H., Thiaville, A., Rohart, S., Fert, A. & Chshiev, M. Anatomy of Dzyaloshinskii-Moriya interaction at Co/Pt interfaces. *Phys. Rev. Lett.* **115**, 267210 (2015).

3. Sampaio, J. *et al*, stability and current-induced motion of isolated magnetic skyrmions in nanostructures. *Nature nanotechnology* **8**, 839-844 (2013)
